# Changes in soybean cultivars released over the past 50 years in southern Brazil

**DOI:** 10.1038/s41598-021-04043-8

**Published:** 2022-01-11

**Authors:** Renan Caldas Umburanas, Jackson Kawakami, Elizabeth Anna Ainsworth, José Laércio Favarin, Leonardo Zabot Anderle, Durval Dourado-Neto, Klaus Reichardt

**Affiliations:** 1 Luiz de Queiroz College of Agriculture (ESALQ), University of São Paulo (USP), Piracicaba, 13418-900 Brazil; 2 Universidade Estadual do Centro-Oeste (UNICENTRO), Guarapuava, 85015-300 Brazil; 3 Global Change and Photosynthesis Research Unit, USDA ARS, Urbana, 61801 USA; 4 Center of Nuclear Energy in Agriculture (CENA), University of São Paulo (USP), Piracicaba, 13400-970 Brazil

**Keywords:** Plant breeding, Agricultural genetics

## Abstract

On-farm soybean yield has increased considerably in the last 50 years in southern Brazil, but there is still little information about how selection and breeding for yield increase has changed the agronomic attributes of cultivars. The objectives of this study were to evaluate the changes in soybean yield, seed oil and protein concentration, and changes in plant attributes that might be associated with yield improvement of 26 soybean cultivars released over the past 50 years in southern Brazil, sown simultaneously in a common field environment for two growing seasons. The average rate of yield gain was 45.9 kg ha^−1^ yr^−1^ (2.1% ha^−1^ yr^−1^), mainly due increased seed number per area and harvest index. Over year of cultivar release, cultivars became less susceptible to lodging, as well as plant mortality reduced. Meanwhile, the seed oil concentration increased, and seed protein concentration decreased, which could have negative consequences for soybeans use and requires further attention for breeding of future cultivars. Breeders have successfully contributed to the annual rate of soybean yield increase in southern Brazil. By our results, as well as the official on-farm production data, there is no evidence of soybean yield reaching a plateau in the near future in southern Brazil.

## Introduction

Brazil produced 110 million tons of soybeans and exported 58 million tons on average from the 2015/2016 until 2017/2018 growing season, making it the second largest producer and the largest soybean exporter in the world^1^. Considering this period, the three largest soybean producing states are Mato Grosso (midwestern region, 27%), Paraná (southern region, 16%) and Rio Grande do Sul (southern region, 16%)^2^.

In the last 42 years, soybean production in Brazil increased 9.4-fold, as a result from both a 5.2-fold expansion in production area and a 1.8-fold increase in on-farm yield, reaching an average yield gain of 43.9 kg ha^−1^ yr^−1^^2^. This improvement in yield has been associated with the continual introduction of new soybean cultivars and with improvements in management practices. Although being one of the world's largest soybean producers, there is still little information about how local selection for greater yields has changed the plant attributes of soybean cultivars in this region.

Soybean cultivation has been reported in Brazil since 1882, but the crop became economically important around 1970^3^. In this context, the introduction of cultivars in the southern part of the country can be divided into two phases: (i) introduction of cultivars from southern United States into regions below 23° S, pioneering soybean production of this region; and (ii) introduction of transgenic cultivars with indeterminate growth habit adapted to no-tillage management practices in the 2000s^3^. Many of these introduced transgenic cultivars had early maturity and came from Argentinean soybean breeding companies.

The changes in the soybean plant that led to increased yield is a topic under constant study in the different producing countries. There are previous studies reporting such changes from Argentina^4^, United States^5–8^, China^9–11^, Canada^12^, and India^13^.

Previous studies with soybean cultivars developed and introduced in Brazil that tested genetic gain used experimental soybean lines from breeding programs released until the year 2000 or earlier^14–16^, and there is only one published recently with a long-term historical set of 29 cultivars^17^, but none of these studies evaluated the concentration of oil and protein. For United States and Canada, breeders increased soybean yields by significantly increasing harvest index, canopy light interception and energy conversion efficiency^6,12^. In China, yield increases were primarily associated with heavier 100-seed weight and number of seeds per plant^11^. In India and Argentina, the increase of number of seeds per area increased with cultivar year of release while 100-seed weight showed no significant trend^4,13^. These studies show evidence for regional variation in attributes that contributed to yield gain.

The genetic gain analysis enables comparison of the gains obtained in different environments or with the use of different breeding strategies^18^. Evaluating key attributes that increase soybean yield over time is strategic for planning yield increases in future cultivars. From the 2015/2016 until 2017/2018 growing season, the average soybean yields obtained for Brazil, southern Brazil and the Paraná state were similar: around 3.3, 3.3 and 3.4 Mg ha^−1^, respectively^2^. These averages are close to those reached in the United States, 3.4 Mg ha^−1^ and slightly higher than those reached in Argentina, 2.8 Mg ha^−1^, respectively^1^. In the last growing seasons, the national soybean yield contest reached yields of 7.1 to 8.9 Mg ha^−1^ with national champions commonly from the southern part of the country, especially from Paraná state^19^. Such results obtained in soybean yield contest show that there is a room to double the present Brazilian soybean yield by adopting the technology already available to the farmers^20^.

The objectives of this study were to evaluate the changes in soybean seed yield, and seed oil and protein concentration from 26 Brazilian soybean cultivars released over the past 50 years of cultivation. Changes in plant attributes associated with yield and seed constituents were examined since the cultivars were now grown simultaneously in a common field environment for two growing seasons in southern Brazil.

## Results

**Figure 1:**
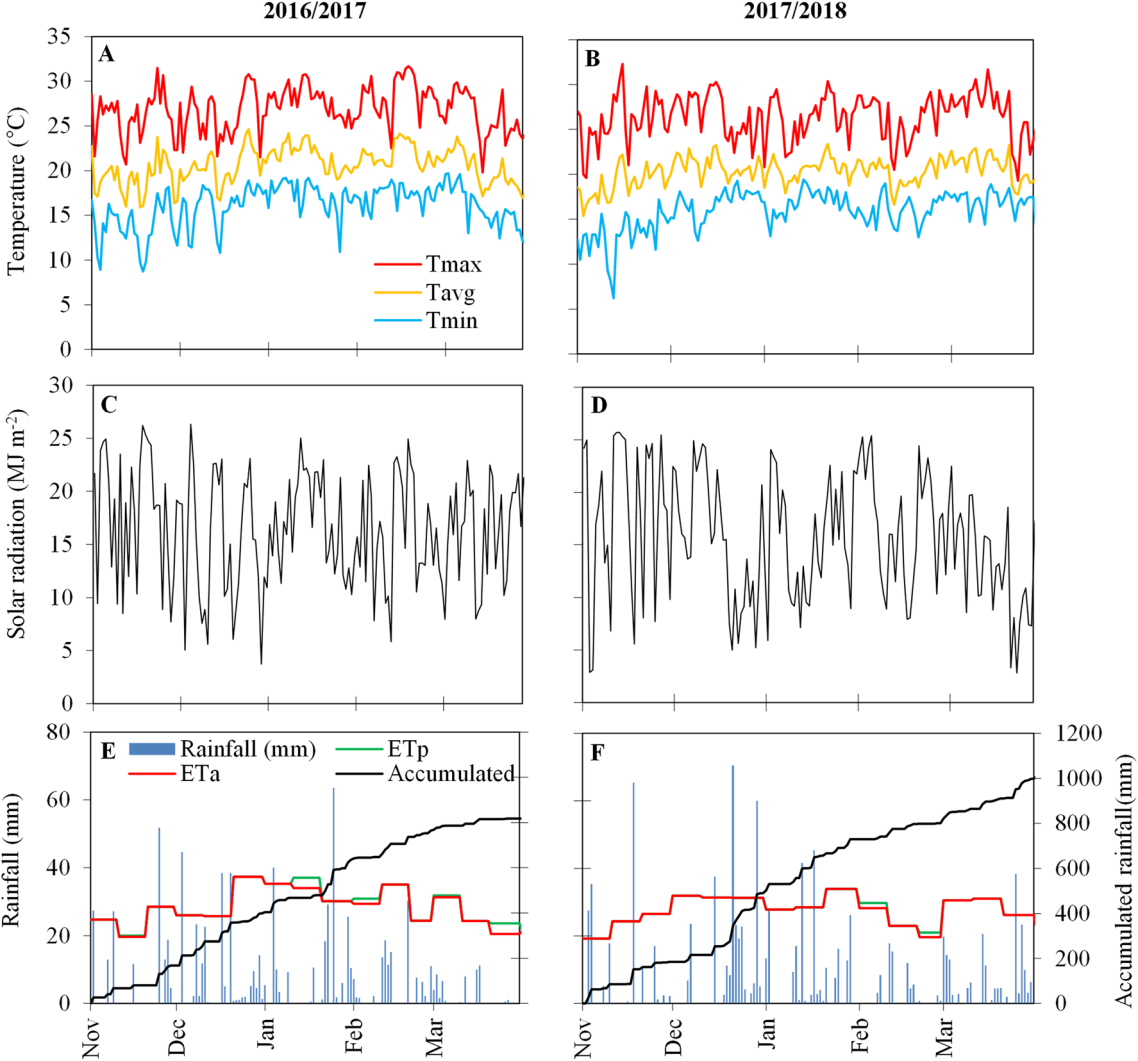
Meteorological data for the 2016/2017 and 2017/2018 growing seasons (from sowing date until 30 Mar): daily maximum (T_MAX_, red), average (T_AVG_, orange), and minimum (T_MIN_, blue) temperatures (**A**,**B**), daily total solar radiation (**C**,**D**), and rainfall (blue bars), accumulated rainfall (black line), potential evapotranspiration (ETp, green line) and actual evapotranspiration (ETa, red line) across the growing season (**E**,**F**).

During crop growth, the mean temperature was similar in the two growing seasons (20.6 and 20.3 °C) (Fig. 1A,B). Accumulated solar radiation was also similar in the two growing seasons (2584 and 2537 MJ m^−2^; Fig. 1C,D). For southern Brazil, rainfall accumulation around 800 mm is enough to maximize soybean yield^21^. Cumulative rainfall was 823 mm in 2016/2017 and 984 mm in 2017/2018 (Fig. 1E,F). The actual and potential evapotranspiration was close in the two growing seasons, indicating that the soybeans did not suffer from water deficiency.

### Relationship between grain yield and year of cultivar release

**Figure 2:**
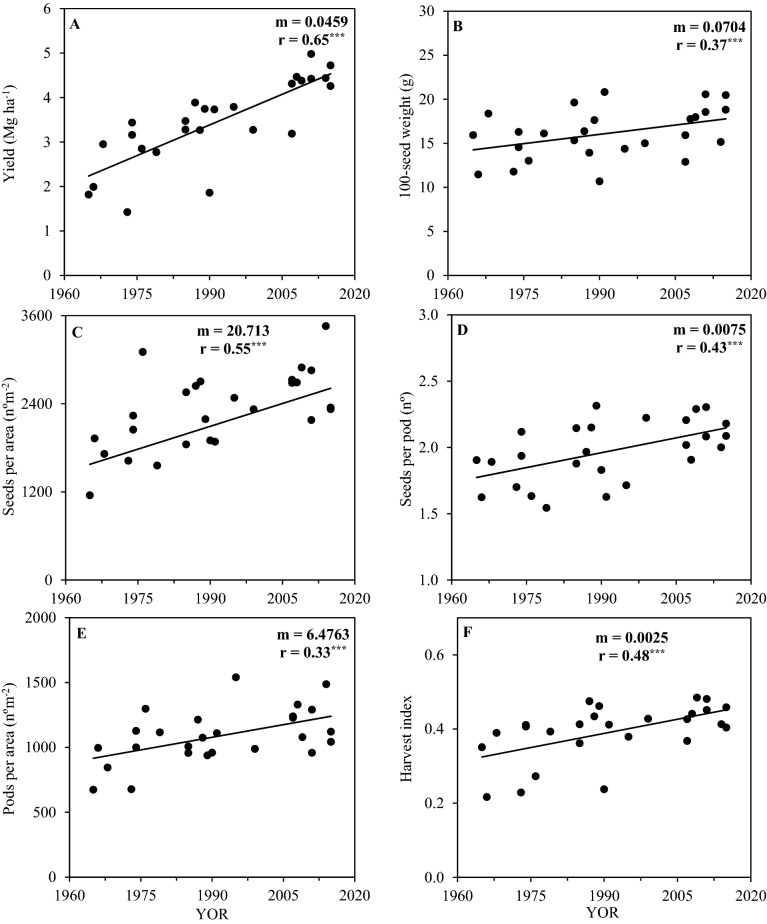
Seed yield (**A**), 100-seed weight (**B**), seeds per area (**C**), seeds per pod (**D**), pods per area (**E**) and harvest index (**F**) with year of release (YOR) of 26 soybean cultivars evaluated in the 2017 and 2018 harvest seasons. Black lines represent significant linear regression. *m*, slope; *r*, Pearson correlation coefficient; ns, non-significant; ^*^ *p* ≤ 0.05; ^**^ p ≤ 0.01; and ^***^ *p* ≤ 0.001.

The yield among the 26 evaluated soybean cultivars increased significantly with year of release (YOR) and varied from 1.6 to 5.7 Mg ha^−1^ in 2017 and from 0.7 to 5.3 Mg ha^−1^ in 2018 (Fig. 2A). The average yield gain by YOR was 45.9 kg ha^−1^ yr^−1^ (2.1% ha^−1^ yr^−1^), i.e., yield increased 2.3-fold from 1965 to 2015.

### Relationship between plant attributes and year of cultivar release

In both seasons the increasing yield trend with YOR was associated with more seeds per area as consequence of more pods per area and more seeds per pod, besides a slightly greater 100-seed weight. The 100-seed weight was positively correlated with the soybean cultivar YOR in both growing seasons, increasing by 70.4 mg yr^−1^ (Fig. 2B). The number of seeds and pods per area were also positively correlated with soybean cultivar YOR in both growing seasons, increasing by 20.7 seeds per m^2^ and 6.5 pods per m^2^ (Fig. 2C,E). The number of seeds per pod also increased with YOR by 0.0075 seeds pod^−1^ yr^−1^ (Fig. 2D). Harvest index increased with YOR in both growing seasons by 0.0025 units per year (Fig. 2F).

**Figure 3:**
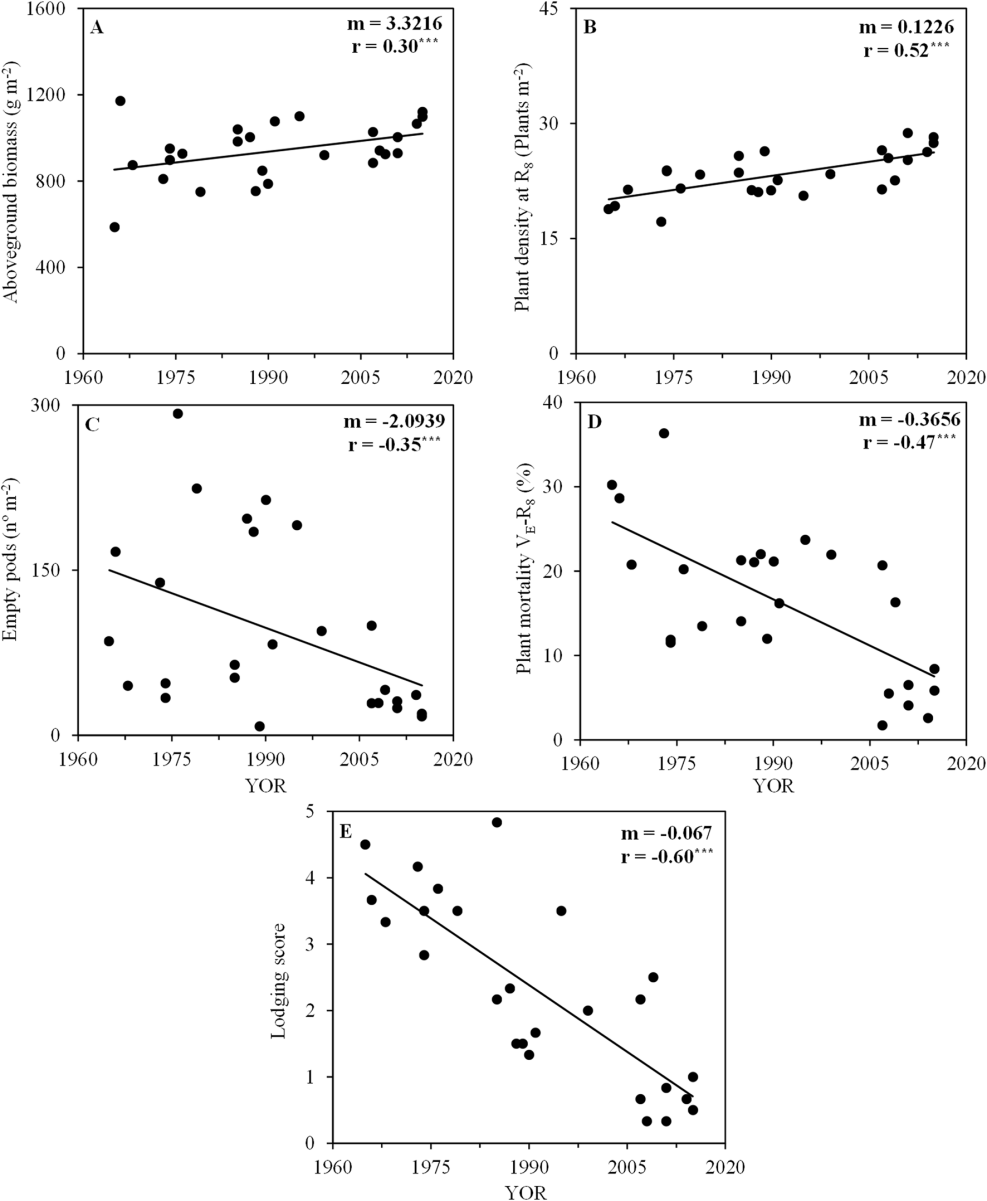
Aboveground biomass at R_5_ (**A**), plant density at harvest (R_8_) (**B**), number of empty pods per area (**C**), plant mortality from emergence (V_E_) to harvest (R_8_) (**D**) and lodging score (**E**) with year of release (YOR) of 26 soybean cultivars evaluated in the 2017 and 2018 harvest seasons. Black lines represent significant linear regression. *m*, slope; *r*, Pearson correlation coefficient; ns, non-significant; and ^***^ *p* ≤ 0.001.

The aboveground biomass was positively correlated with YOR in both growing seasons, increasing by 3.32 g m^−2^ yr^−1^ (Fig. 3A). The remaining plant density at full maturity (R_8_) had a positive correlation with YOR, in both growing seasons (Fig. 3B), while plant mortality from emergence to harvest decreased over YOR, i.e., more plants that emerged reached full maturity (Fig. 3D). The number of empty pods was negatively correlated with YOR in both growing seasons, reaching values close to 0 in the most modern cultivars (Fig. 3C). Lodging scores also decreased with YOR by 0.07 units per year (Fig. 3E).

**Figure 4:**
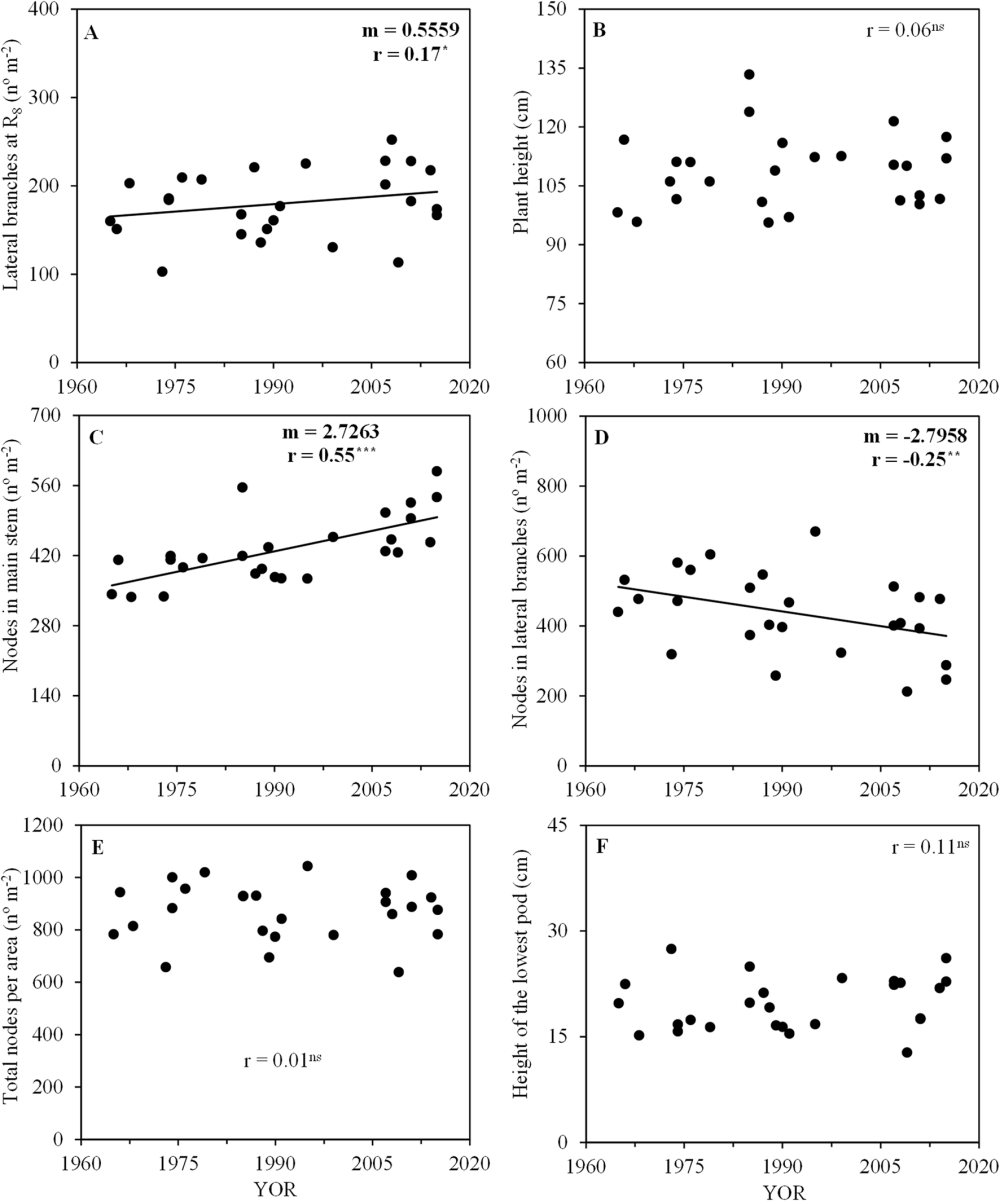
Lateral branches (**A**), plant height (**B**), node number on the main stem (**C**), node number on lateral branches (**D**), total nodes per area (**E**), and height of the lowest pod (**F**) with year of release (YOR) of 26 soybean cultivars evaluated in the 2017 and 2018 harvest seasons. Black lines represent significant linear regression. *m*, slope; *r*, Pearson correlation coefficient; ns, non-significant; ^*^ *p* ≤ 0.05; ^**^ *p* ≤ 0.01, and ^***^ *p* ≤ 0.001.

The number of lateral branches per area at harvest was positively correlated with YOR increasing by 0.56 branches per m^2^ yr^1^ (Fig. 4A). Plant height was not significantly correlated with YOR (Fig. 4B). The node number on the main stem per area presented a positive correlation with YOR (Fig. 4C). The node number on lateral branches per area decreased with YOR by 2.80 nodes m^−2^ yr^−1^ (Fig. 4D). The number of nodes per area and the height of the lowest pod showed no consistent trend over the YOR (Fig. 4E,F).

**Figure 5:**
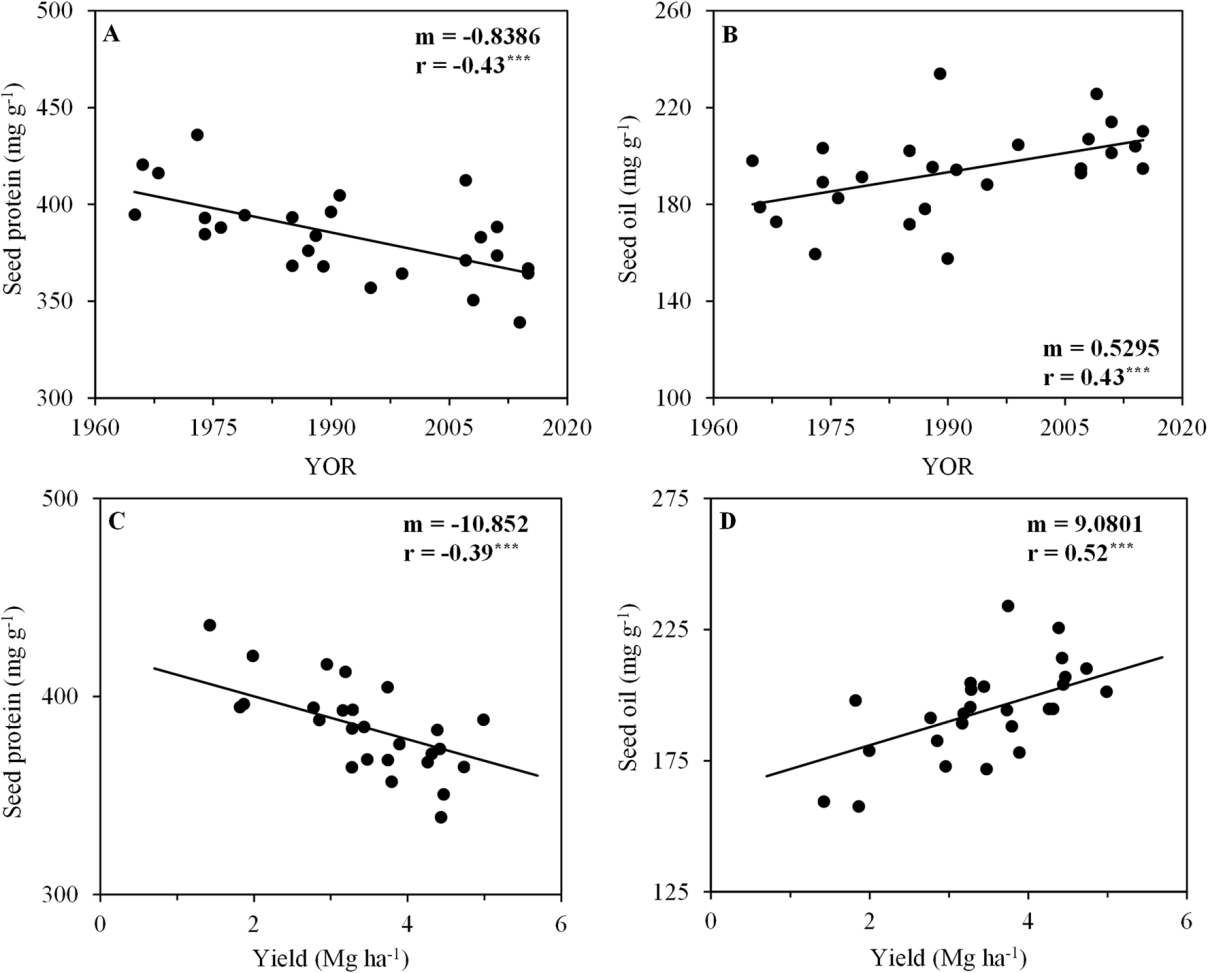
Correlations between seed protein concentration (**A**,**C**) and seed oil concentration (**B**,**D**) with year of release (YOR) and with yield of 26 soybean cultivars evaluated in the 2017 and 2018 harvest seasons. Black line represents significant linear regression. *m*, slope; *r*, Pearson correlation coefficient; ns, non-significant; and ^***^ *p* ≤ 0.001.

The seed protein concentration showed a negative correlation with soybean cultivar YOR, decreasing by 0.84 mg g^−1^ yr^−1^ (Fig. 5A). The seed oil concentration was positively correlated with soybean cultivar YOR, increasing by 0.53 mg g^−1^ yr^−1^ (Fig. 5B).

### Relationships between plant attributes and yield

Seed protein concentration was negatively correlated with yield (Fig. 5C), while seed oil concentration was positively correlated with yield (Fig. 5D).

**Figure 6:**
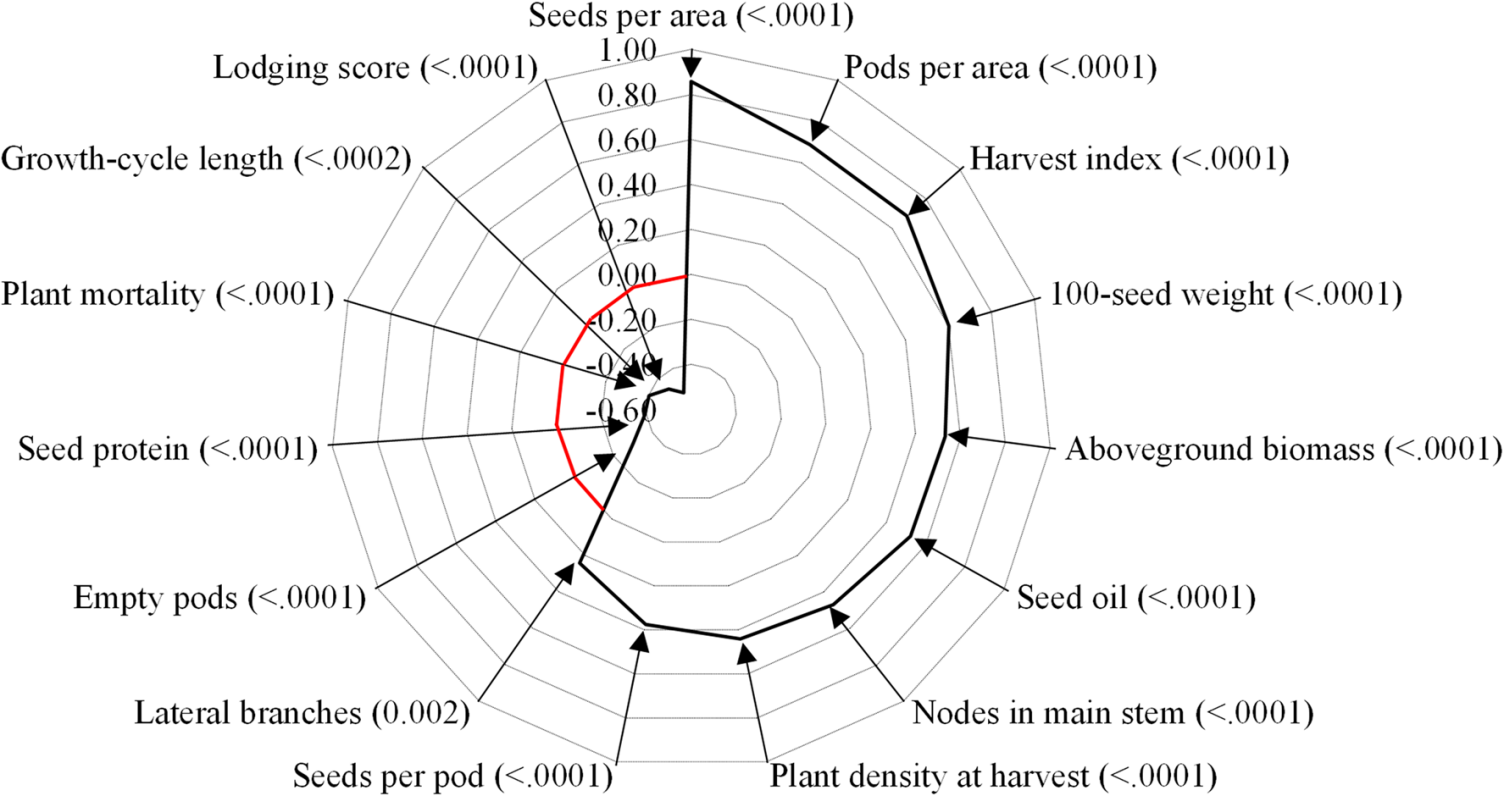
Pearson correlation between agronomic attributes and yield of 26 soybean cultivars released in the last 50 years in southern Brazil. Each ring represents a level of significance by the Pearson correlation, ranging from 1 to -0.8. In parentheses are the significance (*p*) accounting for the 2017 and 2018 harvest seasons. The arrows indicate the correlation points of each attribute. The red represents zero correlation.

For both growing seasons grain yield was positively correlated with seeds per area, pods per area, harvest index, 100-seed weight, aboveground biomass, seed oil concentration, nodes in main stem, plant density at harvest, seeds per pod and number of lateral branches. Yield was negatively correlated with lodging score, growth-cycle length, plant mortality, seed protein concentration, and empty pods per area (Fig. 6). Nodes in lateral branches, total nodes per area, height of lowest pod, and plant height had no consistent correlation with grain yield over the growing seasons (Fig. 6).

## Discussion

In the past 50 years of soybean cultivation in Brazil, the average on-farm yield has increased in a linear fashion^1^. Our evaluation of old and modern soybean cultivars in the same environment showed that yields increased by 45.9 kg ha^−1^ yr^−1^ (Fig. 2A) in two harvest seasons, which was similar to the average on-farm yield gain obtained in Brazil, southern Brazil and Paraná state of 44, 43 and 39 kg ha^−1^ yr^−1^, respectively^2^. This shows that the release of improved cultivars has been a substantial driver for on-farm yield increases.

**Figure 7:**
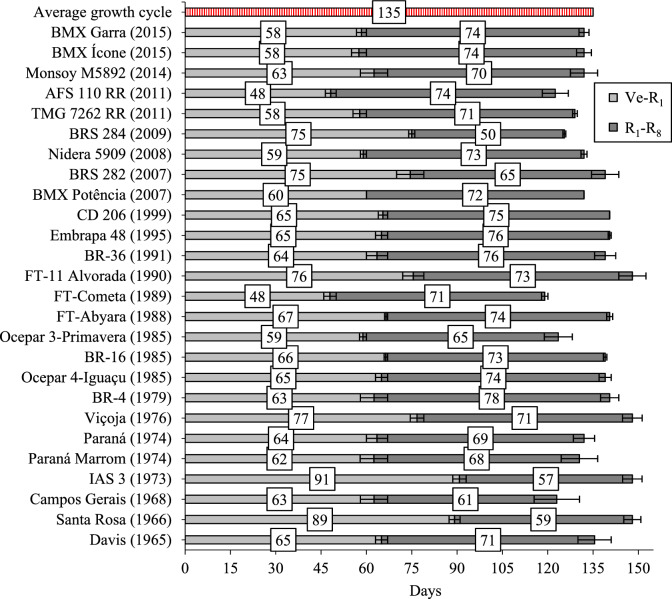
Time between emergence and beginning of flowering (V_E_-R_1_), and between beginning of flowering and physiological maturity (R_1_-R_8_) of 26 soybean cultivars released over the past 50 years in the average of the 2017 and 2018 harvest seasons. Bars represent the average deviation.

In this study, old and modern soybean cultivars were evaluated in the context of current agronomic practices, since in order to determine the genetic basis for increased yield, cultivars must be evaluated in a common environment^7,22^. Modern cultivars tend to have a shorter vegetative growth period (V_E_–R_1_) compared to older cultivars (Fig. 7).

As management practices evolved over time along with the release of newer cultivars, the rate of on-farm yield increase is a result of the interaction between the cultivar and the environment. More studies are necessary to separate the effects of management from the effects of cultivar on increased on-farm yield; and to understand how older cultivars from Brazil perform under less favorable conditions that occurred more often in the past, e.g., low fertility, acid soils, or high aluminum concentrations in the soil. Modern cultivars from United States produce more than the older ones even in low yield environments, but especially in high yield environments^7^. For cultivars and environmental conditions from Brazil this is unknown and further studies with a historical set of cultivars under less favorable conditions would be needed to test the interaction.

The rate of yield gain in cultivars from southern Brazil is similar to those obtained for cultivars from Argentina (43 kg ha yr^−1^)^4^. Previous studies carried out in Paraná, southern Brazil, with soybean lines released from 1981 to 1986 indicated yield increases of 45 and 37 kg ha^−1^ yr^−1^ for early and semi-early maturity cultivars, respectively^14^. More recently, genetic gains of 39.4 and 40.7 kg ha^−1^ yr^−1^ were reported^17^. Several studies from major soybean producing countries have also reported yield gains, with different sets of historical cultivars and maturity groups (Table 1).

**Table 1:** Summary of the soybean yield gain results, authors, country, environments (E) and/or seasons (S), latitude, years of breeding period (BP), maturity group (MG), number of cultivars (n), and yield gain (YG).

Authors	Country	E/S	Latitude	BP	MG	n	YG (kg ha^−1^ yr^−1^)
Boehm Jr. et al. (2019)^23^	USA		–	80	V to VII		13.7
Fox et al. (2013)^5^	USA	6	From 41.8°N to 37.4°N	85	II to IV	130	22.8
Koester et al. (2014)^6^	USA	1	40°N	84	–	24	26.5
Rincker et al. (2014)^7^	USA	41	–	85	II to IV	168	29
Suhre et al. (2014)^24^	USA	4	From 44°N to 40.3°N	80	II and III	116	19.3 to 24.1
Rowntree et al. (2014)^25^	USA	3	43.3°N; 40°N 40.3°N	85	II and III	116	19.8
Rogers et al. (2015)^8^	USA	3	–	80	IV to VI	45	16.8
Kahlon and Board (2012)^26^	USA	1	30°N	46	V to VIII	18	30.7
Morrison et al. (2000)^12^	Canada	1	45.4°N	58	–	14	10.2
Kumudini et al. (2001)^27^	Canada	1	43.6°N	60	–	4	18
Jin et al. (2010)^9^	China	1	47.4°N	56	00 and 0	45	10.1
Liu et al. (2012)^28^	China	1	43.5°N	82	–	38	12.5
Wu et al. (2015)^29^	China	9	From 50.1°N to 42.3° N	84	–	64	6 to 16
Wang et al. (2016)^29^	China	3	36.7°N to 34°N	80	–	25	9.97
Qin et al. (2017)^11^	China	–	–	60	–	2155	13 to 24
Cui et al. (2016)^30^	China	1	43.5°N	86	–	27	18.9
Ramteke et al. (2011)^13^	India	1	22.1°N	39	–	17	23
de Felipe et al. (2016)^4^	Argentina	1	33º1S	35	III to V	181	43
de Toledo et al. (1990)^14^	Brazil	3	–	5	–	–	37 to 45
Todeschini et al. (2019)^17^	Brazil	2	25.8°S; 26.2°S	46	–	29	39.4 to 40.7
**Umburanas et al**	**Brazil**	**2**	**25.4°S**	**56**	**V to VI**	**26**	**45.9**

The genetic improvement in yield of soybean cultivars from Brazil and Argentina were greater than those reported in the United States, China, Canada, and India. As commercial soybean production is more recent in South America, greater rates of yield increase show that breeders were quick to develop adapted and productive cultivars for Brazil and Argentina, which currently achieve on-farm yield averages similar to those obtained in the United States^1^.

For cultivar breeding, high-yielding environments contribute to maximize the expression of genetic yield potential, even if the yield potential is not reached under farmers' field conditions^7,22^. In addition, the selection of cultivars in unfavorable environments, with lower yield potential, such as low fertility, high weed incidence and so on stressful conditions, helps to identify more resilient cultivars^31^, and it is largely unknown how breeding affected this performance in modern cultivars compared to the older ones, especially the response to high temperatures and drought.

For the cultivars studied, the number of seeds per area, number of seeds per pod, number of pods per area, and 100-seed weight were all correlated to YOR and yield gain (Fig. 6), similar to results from cultivars from China^10,11,29^. However, for soybean cultivars from the United States and Canada, no consistent relationship between soybean cultivar YOR and 100-seed weight were found^7,12,22^. The major driver for soybean yield increases in our study was the number of seeds per area, being consistent with other studies^9,12^. Lodging was reduced over the YOR (Fig. 3E) and plant mortality was also reduced (Fig. 3D), made more plants survive from emergence until harvest (V_E_-R_8_) and with upright canopy (Fig. 3B) which allowed greater formation of pods and seeds per area (Fig. 2C).

The different plant mortalities between cultivars (Fig. 3D) were probably due to differences in canopy architecture. Modern cultivars had more upright canopies as evidenced by the lodging score (Fig. 3E), as well as in the past 50 years of cultivar release the increase in the harvest index was higher (1.52-fold, Fig. 2F) than the increase in aboveground biomass (1.19-fold, Fig. 3A). As diseases and pests were adequately controlled during the study, possibly the reasons for the different lodging score and plant mortality are due to competition within the soybean canopy caused by attributes such as stem strength, root support, and/or canopy weight (excessive vegetative growth), but this should be further evaluated in future studies.

The increase in yield potential in soybean cultivars from United States is associated with more efficient light interception and energy conversion efficiency^6,22^. In modern cultivars from United States the yield is strongly associated with the number of seeds per area and positively correlated with crop growth rate^32^.

The ability to increase the aboveground biomass up to the end of seed filling stage (R_5_) is a critical component for increasing the number of seeds per area, since the seed filling rate as well as the 100-seed weight show little or no variation^32^. Experiments performed with elevated [CO_2_] concentration suggest that the capacity of the source (photoassimilates) is greater than that of the sink (pods and seeds), as soybean cultivars showed increases in leaf photosynthesis of 24%, while yield increased only by 15% and the harvest index decreased^33,34^. In addition, there is significant genetic variation in soybean response to elevated [CO_2_] concentration^35^, so it seems that in the future more productive cultivars need a greater reproductive sink, that is, more nodes, pods, and seeds.

In this study, the height of the lowest pod from both old and modern cultivars was higher than 10 cm and was suitable for mechanical harvest (Fig. 4F). For some cultivars, the height of the lowest pod was around 20 to 30 cm, having infertile nodes in these lower canopy layers. The production of pods and seeds in these nodes could be necessary to improve cultivar yield potential, being a desirable attribute. Soybean cultivars with plant architecture that allow greater light distribution within the canopy have higher yield, mainly due to high pod fixation and seed filling rate^36^.

Aboveground biomass presented a slightly increased trend with YOR in both growing seasons (Fig. 3A). However, some old cultivars, produced as much aboveground biomass than some of the modern cultivars, despite having lower seed yield, which occurred in part due the increase trend in harvest index over YOR (Fig. 2F), and by the longer vegetative growth period in some old cultivars (Fig. 7).

The aboveground biomass positive correlation with YOR (Fig. 3A) and negative correlation with lodging (r: − 0.39, significant at *p* ≤ 0.001, data not shown) suggests that the net canopy photosynthesis of modern cultivars increased, but it should be further investigated. In our study plant lodging started mainly after the flowering (R_1_) stage, when some plants overtopped others. Similar results were found for soybean cultivars from United States, where greater photosynthetic efficiency was achieved by the more upright plants with less susceptibility to lodging^6^.

The number of empty pods decreased with YOR in both growing seasons (Fig. 3C). This shows that modern cultivars have greater capacity to fill seeds and/or that modern cultivars are more efficient at aborting pods that they are not able to fill. In soybeans from the United States, the modern cultivars under low plant densities produce more compensatory yield in lateral branches than older cultivars^24^. In this study, there was a slight trend to reduce the number of lateral branches with YOR, but this needs to be further investigated in lower plant densities.

No-consistent trend was observed between plant height and YOR in our study, although there was a great difference among cultivars (Fig. 4B). Some cultivars from China also did not show any trend in plant height with YOR^11^. However, a negative relationship between plant height and soybean cultivar YOR was observed in studies conducted with cultivars from the United States^5,7,8^, Canada^12^ and China^9^. In the search for cultivars more resistant to lodging, breeders opted for shorter plants in the United States which increase seed production per area and harvest index^8^.

The node number on the main stem had a slight upward trend with YOR (Fig. 4C). This attribute is strongly related to temperature, photoperiod, and the cultivar growth habit^37^. The node number on lateral branches had no consistent trend with YOR over growing seasons (Fig. 4D). The non-significant trend occurred because it is a complex trait and because in our study, we used cultivars with both determinate and indeterminate growth habit to represent the cultivars that predominated over time, as the use of determinate growth habit predominated in the past and indeterminate growth habit increased and prevails in the last 20 years^3^.

The lodging resistance is an attribute that is positively related to YOR and yield in Brazil (Fig. 3E). It corroborates with results obtained with cultivars from Argentina^4^, United States^5–8^, Canada^12^, and China^9,10^. Upright plants are more able to intercept photosynthetically active radiation, especially during the seed filling period^6^. The harvest index consistently increased with soybean cultivar YOR in this study (Fig. 2F), which also occurred in cultivars from the United States^6^ and China^9^.

From this set of cultivars, the seed protein concentration had a negative relationship with seed yield in the average of growing seasons (r: − 0.70, significant at *p* ≤ 0.01) in the same way as it occurred with cultivars from the United States^7^. These information shows that for future cultivars, breeding must address the seed composition desired by the consumer market, or by industry, as it is a value aggregator to soybean production.

Selection for greater yields has come at the cost of seed protein concentration in cultivars from different countries. In our study seed protein concentration reduced at a rate of 0.84 mg g^−1^ yr^−1^ (Fig. 5A) and showed a significant negative correlation with yield (Fig. 5C). A reduction in seed protein concentration was also reported in the United States between rates of 0.16 to 0.22 mg g^−1^ yr^−1^^7^ and of 0.18 to 0.35 mg g^−1^ yr^−1^^8^. In Canada, a reduction in seed protein concentration was also reported at a rate of 0.54 mg g^−1^ yr^−1^^12^. However, in Chinese cultivars, the seed protein concentration was not consistently modified in the modern cultivars in relation to the old ones, although the rate of yield improvement has been smaller in China compared to South and North America^9,11^.

Seed oil concentration increased significantly with soybean cultivar YOR at a rate of 0.53 mg g^−1^ yr^−1^ in this study (Fig. 5D), it also increased in cultivars from China between rates of 0.17 to 0.60 mg g^−1^ yr^−1^^11^, and in cultivars from United States between rates of 0.05 to 0.14 mg g^−1^ yr^−1^^7^ and of 0.09 to 0.27 mg g^−1^ yr^−1^^8^. In Canada, an increase in seed oil concentration was also reported at a rate of 0.45 mg g^−1^ yr^−1^^12^. In another study with cultivars from Northeast China, no increase in seed oil concentration was observed with the YOR, but the cultivars used in the past had already higher seed oil concentration, around 200–220 mg g^−1^^9^.

In the soybean breeding program, there is no unique path to increase yield through trait improvements as there is a complex interaction among traits^29^. Although seed concentration of oil and protein, plant height, and 100-seed weight are important attributes, the primary considerations in the decision to release a new cultivar are yield, days to maturity and lodging^7^.

For the subtropical conditions such of this study, maintaining the plant density that emerged until the harvest period (V_E_ – R_8_) proved to be an important attribute. The sample of cultivars used in this study also evidences that breeding improved the canopy architecture of the most modern cultivars, making them less susceptible to lodging.

## Conclusions

Over the last 50 yr. breeders have successfully contributed to the annual rate of soybean yield increase in southern Brazil. The average yield gain rate evaluated in this study, 45.9 kg ha^−1^ yr^−1^, is close to the on-farm rate of yield increase, showing a similar linear trend, which illustrates the important role of genetic improvement in Brazilian soybean production. Increased seed number per area and harvest index were the main contributors to increased yield for the evaluated cultivars. Plants more resistant to lodging provided a more upright canopy, which increased the number of seeds per area. Also, the reduced lodging over the year of cultivar release while plant mortality also reduced, made more plants survives from emergence until harvest and with upright canopy, which allowed greater formation of pods and seeds per area. The seed oil concentration increased, and seed protein decreased with breeding, which could have negative consequences for the use of soybeans and requires further attention in the development of future cultivars.

## Methods

### Site and experimental design

Two field experiments were carried out during the 2016/2017 and 2017/2018 growing seasons at the *Universidade Estadual do Centro-Oeste* research site, in a subtropical environment located in Guarapuava, Paraná State, Brazil (25° 23′ S, 51° 29′ W, altitude 1029 m). By convention, we adopt the year of harvest to refer to each growing season. The soil in the area is classified as very clayey Oxisol (USDA Soil Survey). The climate of the location is classified as Cfb by Köppen’s climate classification system^38^.

In both growing seasons, predecessor crops followed a common rotation scheme from commercial fields. The experimental site for the 2016/2017 growing season had been cultivated with potatoes in the previous summer and black oat in the winter, in a conservation tillage system. The experimental site for the 2017/2018 growing season had been cultivated with maize in the previous summer and black oat in the winter, in a no-till cultivation system. Based on soil chemical analysis (Table 2), limestone was applied at a rate of 850 kg ha^−1^ and 1000 kg ha^−1^ in the winter season of 2016 and 2017, respectively.

**Table 2:** Chemical soil analysis at depths of 0–0.2 m and 0.2–0.4 m from the experimental field sites in Guarapuava, Parana State, Brazil measured before the 2016/2017 and 2017/2018 growing seasons.

Season	Depth	pH	Organic matter	P	K^+^	Ca^2+^	Mg^2+^	H + Al	Al^3+^	CEC†	SO_4_-S
	m	CaCl_2_	g dm^−3^	mg dm^−3^	-mmol_c_ dm^−3^						mg dm^−3^
2016/2017	0–0.2	5.3	37	35	1.2	36	12	34	0	82	11
	0.2–0.4	4.5	32	5	1.2	21	10	80	1	112	56
2017/2018	0–0.2	4.7	39	39	3.5	33	13	64	2	114	12
	0.2–0.4	4.8	27	4	1.2	19	13	58	0	91	29

*CEC* Cation exchange capacity; Organic matter was determined by the Walkley–Black method; P, K^+^, Ca^2+^, and Mg^2+^ were extracted by ion exchange resin; Al^3+^ was extracted by KCl 1 mol L^−1^; H + Al was extracted by SMP method.

The experimental design consisted of completely randomized blocks with three replications of 26 historical soybean (*Glycine max* (L.) Merrill) cultivars (Table 3). These cultivars, released from 1965 to 2015, were selected based on a survey with farmers, discussion with researchers and from the scientific literature.

**Table 3:** List of soybean cultivars grown with representative period, year of release, cultivar, sowing density (SD), growth habit, and maturity.

Period	Year of release	Cultivar	SD (plants m^−2^)	Growth habit	Maturity
1960 ┤1970	1965	Davis	27	Determinate	Early-season
	1966	Santa Rosa	27	Determinate	Mid-season
	1968	Campos Gerais	27	Determinate	Early-season
1970 ┤1980	1973	IAS3	27	Determinate	Mid-season
	1974	Paraná Marrom	27	Determinate	Early-season
	1974	Paraná	27	Determinate	Early-season
	1976	Viçoja	27	Determinate	Mid-season
	1979	BR-4	27	Determinate	Mid-season
1980 ┤1990	1985	Ocepar 3-Primavera	30	Indeterminate	Early-season
	1985	Ocepar 4-Iguaçu	30	Determinate	Early-season
	1985	BR-16	27	Determinate	Mid-season
	1988	FT-Abyara	27	Determinate	Mid-season
	1989	FT-Cometa	30	Indeterminate	Early-season
1990 ┤2000	1990	FT-11 Alvorada	27	Determinate	Mid-season
	1991	BR36	27	Determinate	Mid-season
	1995	Embrapa 48	27	Determinate	Mid-season (VI)
	1999	CD 206	30	Determinate	Mid-season
2000 ┤2010	2007	BRS 282	27	Determinate	Mid-season (VI)
	2007	BMX Potência	27	Indeterminate	Mid-season (VI)
	2009	BRS 284	27	Indeterminate	Early-season (VI)
	2008	Nidera 5909	27	Indeterminate	Early-season (V)
2010 ┤2016	2011	AFS 110 RR	30	Indeterminate	Early-season (VI)
	2011	TMG 7262 RR	27	Half determinate	Early-season (VI)
	2014	M5892IPRO	27	Half determinate	Early-season (V)
	2015	BMX Ícone	30	Indeterminate	Mid-season (VI)
	2015	BMX Garra	30	Indeterminate	Mid-season (VI)

In the past, the relative maturity of cultivars in Brazil were classified as early, mid, and full-season based on location^39^, but it was not successful in describing maturity for different latitudes and environments that occur in the soybean production region of Brazil^39^. This classification was gradually replaced from the 2000s onwards by the maturity group (MG) method, which groups cultivars based on photoperiod responsiveness and adaptation area, later introduced mainly by foreign soybean breeding companies^40^. During the choice of cultivars, we selected early and mid-season growth cycles between those released before the 2000s, and group V and VI for those launched after the 2000s (Table 3).

Fifteen seeds per old cultivar were obtained from germplasm bank of Brazilian Agriculture Research Corporation and were multiplied prior to sowing to have enough seed number, as well as high seed physiological potential. Modern cultivars were obtained from breeder companies or seed suppliers. The plant experiments were performed in accordance with relevant guidelines and regulations.

Cultivar plots contained 4 rows, each 5 m long with 0.45 m row spacing. The experimental area was limited to the two central rows, excluding 0.5 m from the edges. Before sowing, seeds were treated with Pyraclostrobin [25 g L^−1^], Methyl thiophanate [225 g L^−1^] and Fipronil [250 g L^−1^] at the rate of 2 mL per kg of seeds. On the sowing day inoculation with *Bradyrhizobium japonicum* was performed using turfous inoculant with 2.4 g per kg of seeds containing around 5 billion viable cells per gram of inoculant.

Seeds were sown on November 4 in both 2016 and 2017 in adjacent field areas. This sowing date was within agroclimatic zoning that benefits potential soybean yield in this subtropical environment^21^. Basic fertilizer application consisted of 80 kg ha^−1^ of P_2_O_5_, 80 kg ha^−1^ of Ca, 53 kg ha^−1^ of S (single superphosphate) and 70 kg ha^−1^ K_2_O (potassium chloride).

The seeding rate interval recommended by breeders was different between cultivars and we tried to adjust it within this recommended range, aiming for the best condition for the development of the cultivars. Approximately 44 seeds m^−2^ were sown and were thinned out during the V_C_-V_E_ growth stages^41^ to fit within the recommended plant density (Table 3). Weeds were controlled by herbicide before crop emergence and mechanically removed over the growing season. Pests and diseases were adequately controlled.

### Evaluations

Four plants per plot were harvested at the grain filling (R_5_) growth stage when each cultivar reached this stage. Leaf and stem biomasses were dried in a forced air drier at 60 °C for 48 h.

An area of 3.6 m^2^ per plot was harvested at the full maturity (R_8_) growth stage to determine yield, 100-seed weight, number (nº) of seeds per area, nº of seeds per pod, nº of pods per area, height of the lowest pod, aboveground biomass, plant density at harvest, nº of empty pods per area, plant mortality, nº of lateral branches per area, plant height, node nº on the main stem per area, node n﻿º on lateral branches per area, lodging score, harvest index, seed protein concentration, and seed oil concentration. The 100-seed weight was evaluated from a subsample of 600 seeds. The height of the lowest pod was measured from the first pod insertion point to soil surface. The aboveground biomass used was the sum of dry leaf biomass at R_5_, plus dry biomass of stem, seed, and pod shell at R_8_. Seed moisture was adjusted to 130 g kg^−1^. Plant mortality was assessed by the relationship between plant density at harvest and the initial plant density. Death plants were considered the plants that did not reach full maturity, i.e., produced no seeds or pods at all. The Lodging score was evaluated through visual qualitative evaluation with grades from 0 to 5, where 5 indicates 100% lodging and 0 no lodging at all. Apparent harvest index was determined by dividing seed mass by aboveground biomass at R_8_ (stem, seed, and pod). Seed samples were finely ground using a plant mill prior to seed oil and protein evaluation. Seed nitrogen concentration was determined by indophenol blue spectrophotometric method^42^ after sulfuric acid digestion (digestion block). Seed protein concentration was calculated as seed nitrogen concentration × 6.25 and expressed on a dry weight basis. Seed oil concentration was determined using the Soxhlet extraction technique with the solvent petroleum ether pro analysis (Method 945.16 from AOAC^43^). Seed protein and oil concentrations are expressed on a 130 mg g^−1^ moisture basis.

### Meteorological data

Daily meteorological data including temperature (Fig. 1A,B), solar radiation (Fig. 1C,D), and rainfall (Fig. 1E,F) were obtained from a meteorological station (*SIMEPAR*/Brazil) located around 100 m far from the experiments. A sequential water balance^44^ was calculated to identify phases with water deficit during the crop growing season (Fig. 1E,F).

### Data analysis

Analysis of variance (ANOVA) was determined (Table 4) using a mixed model with cultivar effect considered fixed, with a random intercept for growing season, block, and block within growing season, using the PROC MIXED procedure of SAS On Demand for Academics (SAS Institute, Inc., Cary, NC). The total variation (σ^2^p) for each treatment was partitioned into variance components – cultivar (σ^2^c), growing season (σ^2^gs), and cultivar × growing season interaction (σ^2^c × gs) variance—using the VARCOMP procedure of SAS. As σ^2^c were higher than σ^2^c × gs for the evaluated attributes, data were averaged across growing seasons according to Gomez and Gomez criteria^45^. Linear regression analysis was performed taking grain yield and evaluated attributes as dependent variable and year of cultivar release as independent variable using PROC REG procedure of SAS (Table 4). Each evaluated plant attribute was plotted against the year of cultivar release to illustrate their changes over time. Pearson’s correlations among evaluated attributes by year of release or yield were calculated to establish relationships and the correlations significance was evaluated by the Student t-test (α = 0.05).

**Table 4:** Significance of analysis of variance and linear regression models between each evaluated attribute and year of cultivar release.

Attribute	Unit	ANOVA_[Cultivar]_ (*p*-value)	Adj R^2^	Linear model	*p*
Yield	kg ha^−1^	< .0001***	0.4164	ŷ = 0.0459x – 88.026	** < .0001**
100-seed weight	g	< .0001***	0.1325	ŷ = 0.0704x – 124.01	** < .0001**
Seeds per area	nº m^−2^	0.0015**	0.2928	ŷ = 20.713x – 39,126	** < .0001**
Seeds per pod	nº	< .0001***	0.1829	ŷ = 0.0075x – 12.898	** < .0001**
Pods per area	nº m^−2^	0.0066**	0.1049	ŷ = 6.4763x – 11,809	** < .0001**
Height of the lowest pod	cm	0.1076^ ns^	–	Non-significant	–
Aboveground biomass	g m^−2^	0.0108*	0.0868	ŷ = 3.3216x – 5673.7	**0.0001**
Plant density at harvest	plants m^−2^	< .0001***	0.2772	ŷ = 0.1226x – 220.86	** < .0001**
Empty pods	nº m^−2^	< .0001***	0.1141	ŷ = − 2.0939x + 4264.6	** < 0.001**
Plant mortality V_E_-R_8_	%	< .0001***	0.2180	ŷ = − 0.3656x + 744.28	** < .0001**
Lateral branches	nº m^−2^	0.0036**	0.0226	ŷ = 0.5559x – 926.82	**0.0337**
Plant height	cm	0.0001***	–	Non-significant	–
Nodes in main stem	nº m^−2^	< .0001***	0.3020	ŷ = 2.7263x – 4996.7	< .0001
Nodes in lateral branches	nº m^−2^	0.0019**	0.0558	ŷ = − 2.7958x + 6005	**0.0017**
Total nodes per area	nº m^−2^	0.0125*	–	Non-significant	–
Lodging score	–	< .0001***	0.3548	ŷ = − 0.067x + 135.76	** < .0001**
Harvest index	–	< .0001***	0.2256	ŷ = 0.0025x – 4.6743	** < .0001**
Seed Protein concentration	mg g^−1^	< .0001***	0.1799	ŷ = − 0.8386x + 2054.3	** < .0001**
Seed oil concentration	mg g^−1^	< .0001***	0.1800	ŷ = 0.5295x – 860.34	** < .0001**

Bold values are *p-*value ≤ 0.05.
